# Monitoring Humoral Response Following BNT162b2 mRNA Vaccination against SARS-CoV-2 in Hematopoietic Stem-Cell Transplantation Patients: A Single-Center Prospective Study along with a Brief Review of Current Literature

**DOI:** 10.3390/hematolrep16020022

**Published:** 2024-04-16

**Authors:** John V. Asimakopoulos, Eleni Lalou, George Seferlis, Maria Malliarou, Eliana Konstantinou, Ioannis Drandakis, Ioannis Vasilopoulos, Angeliki N. Georgopoulou, Anastasia Kopsaftopoulou, Alexandros Machairas, Alexia Piperidou, Anestis Karapaschalidis, Maria-Ekaterini Lefaki, Dimitrios Galopoulos, Maria-Panagiota Arapaki, Panagiota Petsa, Ekaterini Benekou, Marina P. Siakantaris, Athanasios G. Papavassiliou, Panagiotis Tsaftaridis, Panayiotis Panayiotidis, Theodoros P. Vassilakopoulos, Angeliki Papapanagiotou, Maria K. Angelopoulou

**Affiliations:** 1 Department of Hematology and Bone Marrow Transplantation, National and Kapodistrian University of Athens, Laikon General Hospital, Aghiou Thoma 17 str, 11527 Athens, Attica, Greece; elenilalou@laiko.gr (E.L.); yorgos.sef@gmail.com (G.S.); eliankon@hotmail.com (E.K.); johnniedrand@gmail.com (I.D.); g_vasilo19@hotmail.com (I.V.); angelikigeorgo@gmail.com (A.N.G.); an.kopsaftopoulou@gmail.com (A.K.); machairasaleks@gmail.com (A.M.); alexia_piper@hotmail.gr (A.P.); anestiskara@hotmail.com (A.K.); mklefaki@gmail.com (M.-E.L.); arapaki_m@hotmail.gr (M.-P.A.); penny-pet@hotmail.com (P.P.); katbenekou@gmail.com (E.B.); siakantaris@gmail.com (M.P.S.); pantsaftar@gmail.com (P.T.); ppanayi@med.uoa.gr (P.P.); tvassilak@med.uoa.gr (T.P.V.); 2 Department of Biological Chemistry, National and Kapodistrian University of Athens, Biochemistry Laboratory, Laikon General Hospital, Aghiou Thoma 17 str, 11527 Athens, Attica, Greece; maria.malliar@gmail.com (M.M.); papavas@med.uoa.gr (A.G.P.); agpana@med.uoa.gr (A.P.)

**Keywords:** autologous transplantation, COVID-19 pandemic, stem-cell transplantation, BNT162b2 vaccine, SARS-CoV-2, hematologic malignancies

## Abstract

Data on antibody response (AR) after vaccination against SARS-CoV2 in hematopoietic stem-cell transplantation setting (HSCT) were initially scarce, mainly due to the exclusion of such patients from approval studies. Shortly after the worldwide application of vaccination against SARS-CoV-2 in vulnerable populations such as patients with hematologic malignancies, limited single-center trials, including HSCT patients, were published. However, there was a great heterogeneity between them regarding the type of underlying malignancy, co-current treatment, type of vaccine, method of AR measurement, and time point of AR measurement. Herein, we present the results of a prospective study on AR after vaccination for SARS-CoV-2 using the BNT162b2 vaccine in a cohort of 54 HSCT recipients—mostly autologous from a single Unit—along with a broad review of the current literature. In our cohort, the AR positivity rate at 1 month was 80.8% and remained positive in 85.7% of patients at 3 months after vaccination. There were only nine non-responders, who were more heavily pretreated and more frequently hypogammaglobulinemic compared to responders. High antibody titers (AT), [AT ≥ 1000 U/mL], were detected in 38.5% and 30.6% of the patients at m_1_ and m_3_, respectively. A significant decline in AT between m_1_ and m_3_ was demonstrated—*p* < 0.0001; median AT_1_ and AT_3_ were 480.5 and 293 U/mL, respectively. A novel finding of our study was the negative impact of IgA hypogammaglobulinemia on response to vaccination. Other negative significant factors were treatment with anti-CD20 antibody at vaccination and vaccination within 18 months from HSCT. Our data indicate that HSCT recipients elicit a positive response to the BNT162b2 vaccine against SARS-CoV-2 when vaccinated at 6 months post-transplant, and vaccination should be offered to this patient population even within the post-pandemic COVID-19 era.

## 1. Introduction

Patients with hematologic malignancies (HM) demonstrate a higher risk of severe COVID-19 disease, reflected in the doubled mortality rate of the unselected HM population compared to the control group (14% versus 7%) in a large population-based study [1]. Based on a large meta-analysis of observational data on HM patients with COVID-19 disease, the overall mortality rate in hospitalized adults is estimated at around 40%, ranging from 23% to 70% in various studies [2]. Moreover, the clinical outcome of hematopoietic stem-cell transplantation (HSCT) recipients is even more unfavorable; the risk of COVID-19 lower respiratory tract disease exceeds 80% with incidence of ICU admission ranging from 20% to 25% and overall mortality >25% [3,4].

HSCT is considered the standard of care for a variety of HM, albeit it is associated with significant secondary immunodeficiency. Thus, prophylactic vaccination for common pathogens is strongly recommended for the protection of this vulnerable population. During the first two years of the COVID-19 pandemic, vaccination has been the most effective measure against SARS-CoV-2 infection due to the limited therapeutic options. However, HSCT recipients have been excluded from the initial vaccination studies, resulting in uncertainty concerning its efficacy in inducing adequate responses. Lower antibody titers have been observed in solid organ transplantation recipients [5] and in patients with chronic lymphocytic leukemia (CLL) treated with anti-CD20 antibodies [6,7], while data regarding outcomes in HSCT recipients are limited.

We here report the results of a prospectively designed study on antibody response to vaccination for SARS-CoV-2 using the BNT162b2 vaccine in a cohort of 54 HSCT recipients from a single Unit.

## 2. Materials and Methods

This monocentric prospective study included HM patients without previously documented SARS-CoV-2 infection having undergone HSCT—autologous (auto-HSCT) or allogeneic(allo-HSCT)—at least 6 months before and within 5 years from the time of vaccination. All patients were vaccinated between March and May 2021 with two doses of the BNT162b2 vaccine (Pfizer–BioNTech COVID-19 vaccine, New York, NY, United States-Mainz, Germany) as part of the National Public Health Organization Vaccination Program in Greece against SARS-CoV-2 disease. Informed consent was given by all participants. Blood samples for antibody titers (AT) were collected before the first dose (m_0_)—to exclude previous asymptomatic infection- as well as at 1 month (m_1_) and at 3 months (m_3_) after the second dose (δ_2_) of the vaccination. The serum was separated and preserved at −20 °C until AT measurement at each time point: AT_0_, AT_1_, and AT_3_.

Serum antibodies against SARS-CoV-2 were measured with Elecsys Anti-SARS-CoV-2 S immunoassay (Roche Diagnostics International AG, Rotkreuz, Switzerland), which uses a recombinant protein representing the receptor-binding domain (RBD) on the S1 subunit of the spike antigen in a double-antigen sandwich assay format. Numeric values ≥0.8 U/mL are interpreted as “positive”, with the analytical range of the assay measuring between 0.4 and 250 U/mL, reaching up to 2500 U/mL for 10-fold diluted samples. Median AT in healthy individuals at 1 month after vaccination is reported to be ≥1000 U/mL, measured by the same method [6].

Seroconversion, i.e., a positive antibody response (AR), was defined as any value ≥0.8 U/mL. Patient and disease characteristics, type and timing of HSCT, ongoing treatment, and laboratory parameters were examined as possible prognostic factors for response to vaccination. Non-parametric tests were used for such comparisons. Related samples Wilcoxon signed rank test were used to examine differences between AT_1_ and AT_3_. Prognostic factor analysis was performed in two ways: firstly, qualitatively for a positive vs. negative AR by Chi-square and secondly, quantitatively with antibody titers as a continuous variable. *p*-values < 0.05 were considered statistically significant, and all *p*-values were 2-sided. Statistical analyses were carried out with IBM SPSS Statistics version 25. *Time interval between HSCT and vaccination* (TITV) was defined as the time between stem cell infusion and δ2. *No treatment* (NT) was defined as not having received any treatment for the underlying HM for a period of more than 6 months before δ2. *Hypogammaglobulinemia* was defined as values below the lower normal limit level for each class of immunoglobulins: IgG < 7 g/L, IgA < 0.7 g/L, and IgM < 0.4 g/L.

## 3. Results

Fifty-four (54) patients were included in this prospective study. Their characteristics are depicted in Table 1. Briefly, their median age was 56 (19–71) years, and 30 were male. There were only four patients who had undergone sibling allo-HSCT; all four of them had acute leukemia: two acute myelogenous and two acute lymphoblastic. The remaining 50 patients (92%) had been subjected to auto-HSCT: 31 for lymphoma (12 for Hodgkin lymphoma (HL), 19 for non-Hodgkin lymphoma (NHL)) and 19 for multiple myeloma (MM). One patient who had undergone autologous HSCT for transformed follicular lymphoma experienced multiple relapses and finally developed secondary myelodysplastic syndrome with monosomy, and seven were receiving azacitidine at the time of vaccination.

Samples were available at both time points, i.e., at m_1_ and at m_3_ post-vaccination, in 47/54 patients (87%), while five patients had measurements at m_1_ only and two patients at m_3_ only. With the cut-off value of 0.8 U/mL for positivity, 42 out of 52 patients (80.8%) and 42 out of 49 patients (85.7%) had a positive AR at m_1_ and m_3_, respectively. Thus, only 19.2% and 14.3% did not elicit any AR at the prespecified time points, respectively.

When focusing on the characteristics of patients with absent AR, we identified 10 patients with undetectable antibodies at m_1_. Among these 10 patients, only 1 converted to positive with high AT_3_ (97.9 U/mL) and was considered as a responder. Another patient developed a very low AT_3_ (1.38 U/mL), which is very close to the cut-off value of 0.8 U/mL and was considered a non-responder; measurement at m_3_ was unavailable for two patients. The characteristics of these nine non-responders are shown in Table 2. The comparison of these non-responders to the remaining patients revealed that the formers were more heavily pretreated (the median number of previous lines of therapy was five vs. three for patients with AR, *p* = 0.05), and they were more frequently hypogammaglobulinemic [7/9 non-responders (78%) vs. 14.7% of responders, *p* = 0.004]. Treatment at vaccination also differed significantly. The distribution of treatment among non-responders vs. responders was the following: 22.2% vs. 59.5% were off any treatment, 0% vs. 26.2% were receiving lenalidomide, 33.3% vs. 47.0% were on anti-CD20 antibody, and 44.4% vs. 9.5% were on other therapy, respectively.

The distribution of AT is shown in Table 3; no patient had detectable antibodies before vaccination. High titers, i.e., AT ≥ 1000 U/mL, were detected in 38.5% and 30.6% of the patients at m_1_ and m_3_, respectively. There was a significant correlation between AT_1_ and AT_3_ (*p* < 0.0001).

**Figure 1:**
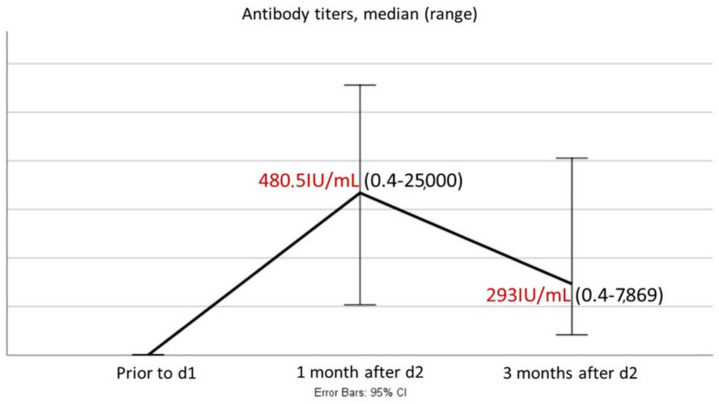
Variability of antibody titers between 1 and 3 months post-vaccination (median AT, 1 and 3 months post-vaccination, are colored with red).

This observation highlights the fact that patients with high AT_1_ did so at m_3_, as well, while patients with lower titers at m_1_ had a similar magnitude of AT_3_. On the other hand, a significant decline in AT between m_1_ and m_3_ was demonstrated, *p* < 0.0001; median AT_1_ and AT_3_ were 480.5 (0.4–25,000) and 293 (0.4–7869)U/mL, respectively (Figure 1).

**Figure 2:**
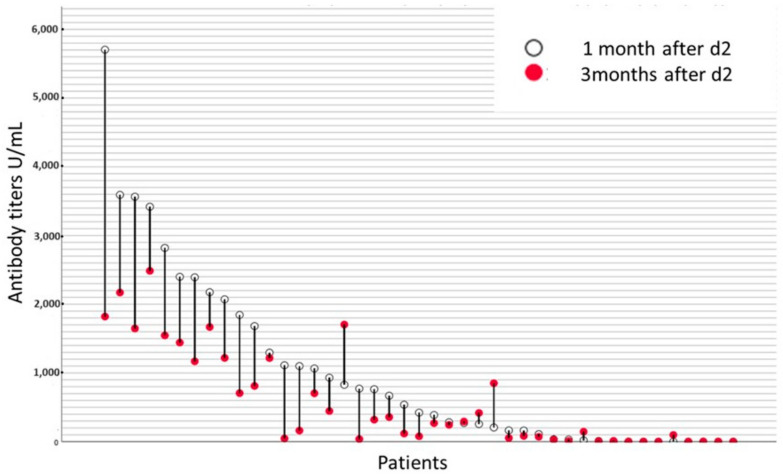
Kinetics of antibody titers between 1- and 3-months post-vaccination at each individual patient.

Specifically, AT decreased in 75% of the patients and increased in 17%, while antibodies remained undetectable in 11% of patients with available both AT_1_ and AT_3_ (Figure 2).

Then, we investigated potential predictive factors for immunogenicity of BNT162b2. We correlated disease and patient parameters firstly with seroconversion (positive AR) and secondly with the magnitude of AR (AT at m_1_ and m_3_). Age, sex, type of HSCT, and absolute lymphocyte/monocyte counts did not prove significant. There was a trend for underlying HM to correlate both with seroconversion and AT (*p* = 0.06 for both): HL patients displayed the highest AT_1_ (median: 2118 U/mL), followed by MM (median: 330.0 U/mL), while NHL patients had the lowest AT (median: 121.15 U/mL). However, most likely, this observation is confounded by the treatment received at the time of vaccination. Interestingly, all four acute leukemia patients who had undergone allogeneic HSCT had high AT (AT_1_ were 538.0 U/mL, 1065 U/mL, 2174 U/mL, and 3591 U/mL, for the four patients).

Factors that proved statistically significant for seroconversion and the magnitude of AT are depicted in Table 4.

### 3.1. Treatment

The most significant factor was the type of treatment at the time of vaccination (*p* < 0.0001). Patients on anti-CD20 antibodies had the lowest AT (median AT_1_ and AT_3_ 0.4 U/mL), followed by those on other chemotherapies (median AT_1_ = 9755 U/mL and AT_3_ = 95.85 U/mL), while the ones on lenalidomide, although positive, they displayed lower AT (median AT_1_ = 210.0 U/mL and AT_3_ = 118.0 U/mL). Patients off any treatment had significantly higher AT: median 1488 U/mL at m_1_ and 1193 U/mL at m_3_. Among the five patients who were on anti-CD20 antibodies at the time of vaccination, three did not elicit any AR and are described in Table 2. One patient had a negative AR at m_1_ and a positive AR at m_3_ (AT_3_ = 97.9 U/mL), and the remaining one had 423.0 U/mL and 77.8 U/mL at m_1_ and m_3_, respectively. Among eight patients receiving other therapies at vaccination, four did not develop any AR and are also depicted in Table 2, while the remaining four had AT ranging between 18.5 U/mL and 1844 U/mL at m_1_ and between 46.7 and 850.0 U/mL at m_3_. All 11 patients on lenalidomide at the time of vaccination had a positive AR; only one with very low titers (AT_1_ = 10.2 U/mL and AT_3_ = 4.06 U/mL). Additionally, more heavily pretreated patients (≥3 lines of previous therapies) had significantly lower AT, both at m_1_ and m_3_, *p* = 0.016 and *p* = 0.026, respectively. However, the seroconversion rate did not differ significantly according to the number of previous lines of treatment.

### 3.2. Hypogammaglobulinemia

Low IgG levels, either at the threshold of 7 g/L or 5 g/L, did not reach statistical significance. Surprisingly, IgA hypogammaglobulinemia predicted both for a lower seroconversion rate at m_1_ and m_3_, as well as for significantly lower AT at both time points. More specifically, 50% and 60% of patients with IgA < 0.7 g/L had a positive AR at m_1_ and m_3_ vs. 86% and 89% for patients with normal IgA levels, respectively (*p* = 0.019 and *p* = 0.046). Median AT at m_1_ for IgA hypogammaglobulinemic patients was 5.3 U/mL vs. 388.0 U/mL for those with normal IgA levels (*p* = 0.02). This difference remained significant at m_3_ with corresponding values of 25.38 U/mL and 269.0 U/mL). Due to this unexpected finding, we further investigated the impact of “any class hypogammaglobulinemia” (IgG and/or IgA and/or IgM levels lower than normal) vs. no hypogammaglobulinemia (IgG and IgA and IgM within normal range). We found that hypogammaglobulinemia of any class was significantly associated with decreased seroconversion rate and lower AT. More specifically, 16/25 patients (64%) with any class hypogammaglobulinemia developed a positive AR at m_1_ vs. 16/17 (94%) among those with normal immunoglobulins of all classes (*p* = 0.02). Moreover, the median AT_1_ was 110 U/mL for those with any class hypogammaglobulinemia vs. 1065 U/mL for the ones with normal immunoglobulin levels (*p* = 0.018). The corresponding AT_3_ values were 86.7 U/mL vs. 850.0 U/mL, *p* = 0.017.

Based on the above observations and in order to further elucidate the role of IgA hypogammaglobulinemia, we identified three groups of patients: *group A*: patients with normal levels of all classes of immunoglobulins; *group B*: patients with normal IgA levels and lower than normal levels of the other two classes of immunoglobulins (either IgG or IgM or both); *group C*: IgA hypogammaglobulinemia, irrespectively of the values of the other two immunoglobulin classes. Group A (*n* = 15) depicted the highest AT_1_, followed by group B (*n* = 14), while group C (*n* = 10) had the lowest AR; AT_1_ was 539.0 U/mL vs. 277.0 U/mL vs. 5.3 U/mL, respectively (*p* = 0.049). The corresponding values at m_3_ post-vaccination were 704.0 U/mL, 203.0 U/mL, and 25.3 U/mL for groups A, B, and C, respectively.

### 3.3. Time between HSCT and Vaccination

Patients vaccinated within 18 months post-HSCT had lower AT at m_1_ compared to the ones who were vaccinated ≥18 months after HSCT, *p* = 0.037, but at m_3_, this difference was not significant (*p* = 0.29).

## 4. Discussion

Recent data on COVID-19 disease in HSCT recipients indicate poor short-term outcomes in both autologous and allogeneic settings; 30-day overall survival after COVID-19 diagnosis was estimated at around 67–68% [3]. Factors such as older age, poor performance status, comorbidities, and high-level immunosuppression were associated with increased mortality [4]. Thus, preventing COVID-19 disease by vaccination of HSCT recipients against SARS-CoV-2 is of uttermost importance. Conditioning regimens, maintenance therapies after transplantation—such as anti-CD20 antibodies, immunomodulatory drugs, and FLT3 inhibitors, presence and treatment of graft-versus-host disease, and persistent hypogammaglobulinemia are the main factors negatively affecting immune response following vaccination of HSCT recipients [4,8].

In August 2021, the CDC’s primary recommendation on booster doses in immunocompromised patients included HSCT recipients within the last 2 years or those under immunosuppressive treatment (https://www.cdc.gov/coronavirus/2019ncov/vaccines/recommendations/immuno.html, accessed on 1 September 2021). However, this recommendation was mainly based on a small case series of solid-organ transplant recipients [8]. More recently (March 2022), ASH and ASCT recommendations strongly recommended vaccination for HSCT recipients, their caregivers, family, and household contacts. Despite that, objective data still remain scarce regarding the type of transplant, time of vaccination, and efficacy of different vaccines, as well as the duration of the immune response.

Table 5 summarizes single-center studies that examined the serologic response of two doses of the BNT162b2 (Pfizer) vaccine against SARS-CoV-2 in HSCT recipients [9,10,11,12,13,14,15,16,17,18,19,20,21,22,23,24,25,26]. The time point of AR measurement varied between these studies: the median time of AT measurement ranged between 18 and 38 days post δ2. They mainly include allo-HSCT recipients, and the positivity rate for AR ranges between 55% and 96%. In addition, most of them provide no information about AT. The threshold of “protective” titers has not been established and differs according to the methodology used, the timing of measurement, and the characteristics of the population studied.

The novelty of our study relies on the fact that this was a prospectively designed study, with all patients having been treated in a single Transplantation Unit and, most importantly, measurements having been performed at pre-specified time points. Moreover, our study included two sequential prospectively defined measurements at 1 and 3 months post δ2, trying to elucidate the short-term kinetics of antibody responses in transplanted patients. Only Tamari et al. have analyzed AT in HSCT recipients at two time points [25].

Data on AR after vaccination with the BNT162b2 vaccine against SARS-CoV2 in auto-HSCT recipients are limited. There are seven other studies with a number of auto-HSCT patients ranging between 38 and 86 [9,10,13,14,20,23,25]. Our study included 54 patients, among whom the majority (50 patients) had undergone auto-HSCT. Although all four patients who had undergone sibling allo-HSCT for acute leukemia had high AT, we cannot draw conclusions regarding allo-HSCT from our data. Thus, our analysis focuses on auto-HSCT.

Our first observation is that >80% of transplanted patients do elicit AR against SARS-CoV-2 after BNT162b2 vaccine. According to a large meta-analysis of 49 studies in adults with HM without allogeneic or autologous HSCT, the pooled AR was 50%, 58%, 61%, and 76% for patients with CLL, aggressive B-NHL, indolent B-NHL, and multiple myeloma [27]. Thus, auto-HSCT per se does not represent a risk factor for blunted AR. Consequently, HSCT recipients should be vaccinated, similarly to the general population. This observation is in accordance with most published studies [9,10,11,12,13,14,15,16,17,18,19,20,21,22,23,24,25,26]. Only two investigators [10,14] reported lower seroconversion rates (60% and 67%). However, both of these analyses were retrospective; different types of vaccines were used, and the time of measurement varied [10,14]. There are only three other studies using exclusively the BNT162b2 vaccine. All of them have reported AR rates of ≥87% [9,13,23], which compares favorably with the 81% and 86% AR rates at 1 and 3 months post-vaccination in our analysis. Furthermore, >30% of our patients had high titers, i.e., ≥1000 U/mL, which has been reported as the median value for healthy individuals at 1 month post vaccination [6]. We chose to include patients who had been transplanted at least 6 months before, according to the usual vaccination programs for other pathogens followed by most HSCT centers [28]. Few other investigators [10,13,23] reported measurement of AT. Comparisons between studies regarding AT cannot be performed due to the different methodologies used. Moreover, the clinical significance of specific AT thresholds is unknown. A drawback of our analysis is the absence of a healthy control (HC) group. Tamari et al., who included an HC group, did find significantly lower AT in auto-HSCT patients compared to HC, while the qualitative seroconversion rate was reported as 100% in HC vs. 87% in the transplant group [25].

Our second observation is the declining trend of AT between m_1_ and m_3_. However, approximately 85% of the patients had a positive antibody response at m_3_. Thus, the usual practice of administering a booster dose between 3 and 6 months after the previous vaccine dose seems rational for HSCT recipients, as well. As mentioned above, only Tamari et al. performed measurements at two time points. Their study was a prospective observational one with a similar design to ours, including 61 autologous recipients. In contrast to our results, they reported a significant increase in AT between the first and the second measurement. However, they chose a different schedule: the first measurement was at 1 month, and the second measurement was at 3 months post-first dose, while we measured AT at 1 and 3 months post-second dose [25]. Practically, both chosen time points of measurement were approximately 1 month earlier than in our analysis, and this difference might explain the contradiction in AT kinetics between their study and ours.

Our third observation is that specific factors may predict the magnitude of AR in HSCT recipients. We found that treatment at vaccination, ≥3 lines of previous therapy, hypogammaglobulinemia, and an interval of <18 months between vaccination and HSCT predict a blunted AR.

Active disease-oriented treatment, especially within 6 months before vaccination has been reported as a negative factor for response by other investigators, as well [13,23]. We found that patients of any treatment had the highest positivity rate (92%) and the highest AT compared to all others on any kind of treatment within 6 months. Rituximab-treated patients demonstrated the lowest AR: among our five patients who had received rituximab, three patients were negative, one patient was negative at m_1_ but seroconverted at m_3_, while only one patient elicited a positive antibody response at both time points. It is widely known from B-NHL and CLL patients that B-cell lymphodepleting treatment with anti-CD20 antibodies is a major determinant of a diminished antibody response [7]. In the auto-HSCT setting, anti-CD20 treatment is associated with >50% failure in seroconversion rate [13,23]. The largest study including rituximab-treated patients by Auttore et al., specifically, demonstrated that those treated with Rituximab within 6 months from vaccination had an inferior AR than those treated >6–≤12 months and >12 months; 13% vs. 50% vs. 87%, respectively [10]. In their study, similarly to ours, the distribution of lymphoma and myeloma patients was well balanced in contrast to others that included more myeloma patients and consequently did not find rituximab as a significant factor [10,20,23]. Other specific treatments that have been associated with a blunted AR are daratumumab [25] (Tamari) and steroids [20]. In our patient population, lenalidomide maintenance did not have an impact on the seroconversion rate but was associated with lower AT compared to any treatment group. A similar observation was reported by Tamari et al. [25]. In our analysis, although the number of previous therapies did not correlate with seroconversion failure, we found that patients who had <3 lines of treatment had significantly higher AT at both time points. Such a correlation has not been reported yet by others. With respect to the TITV, two other studies have identified a time interval of <12 months as a negative predictive factor [9,25]. In our analysis, patients with TITV <18 months elicited significantly lower AT at 1 month compared to those with TITV ≥18 months, but the seroconversion rate was similar.

Another reported predictive parameter for blunted AR is an absolute number of circulating CD19+ B-cells <50/μL at vaccination [25] or ≤100/μL at 30 days post-vaccination [13].

Our most important and novel observation is the impact of IgA hypogammaglobulinemia on response to vaccination. Most studies on hypogammaglobulinemia and anti-SARS-CoV-2 vaccination have generally focused on IgG levels. Thus, IgG hypogammaglobulinemia (<5 g/L) has been identified as a risk factor for blunted AR [7,25]. On the other side, IgA response has been shown as an important factor of early neutralization of SARS-CoV-2 virus after infection, while there is limited data showing both increased positivity for anti-SARS-CoV-2 IgA in the serum and mucosal secretions after immunization [29]. In the HSCT setting, only Tamari et al. reported an inferior AR for patients with IgG levels <5 g/L; however, this was at a non-significant level [25]. No other investigator has analyzed immunoglobulin levels in the auto-HSCT setting. We performed a detailed analysis of the effect of immunoglobulin levels of all classes and showed that hypogammaglobulinemia of any class (IgG, IgA, or IgM) was associated with significantly lower AT. Most importantly, IgA below the lower normal limit proved to be even more significant. Thus, 50% and 40% of patients with IgA <0.7 g/L had a negative AR at m_1_ and m_3_, respectively. Moreover, AT was significantly lower at both time points for IgA hypogammaglobulinemic patients. Based on these findings, we further investigated the impact of IgA hypogammaglobulinemia in combination with abnormalities of other classes of immunoglobulins. We identified three groups of equally distributed number of patients. Patients with normal levels of all immunoglobulin classes displayed the highest AT; those with normal IgA levels but low levels of either/and IgG or IgM had the second highest AT, while the ones with lower-than-normal IgA irrespectively of IgG and IgM levels had the lowest AT. This observation highlights the importance of IgA levels in eliciting proper response to vaccination. Thus, measuring not only IgG but also IgM and IgA levels is essential for predicting HSCT recipients with inadequate AR.

We identified nine patients—16.6% of the whole patient population—who had absent AR. These non-responders differed from responders in many aspects: they were more heavily pretreated, and almost 80% were hypogammaglobulinemic vs. ~15% of responders. Additionally, approximately 80% of non-responders were on active treatment at vaccination, in contrast to 40% of responders.

Regarding the limitations of our study, it should be noted that it included a relatively small number of patients. Secondly, we have not examined the B-cell and T-cell subpopulations of these patients. Moreover, our study lacks analysis of other cellular immunity markers and especially lacks the measurement of neutralizing antibodies against SARS-CoV-2. Lastly, our study did not include a healthy control group AR in order to be compared with HSCT recipients, and it lacks long-term measurement of AR of these patients in order to identify the durability of their response.

HSCT recipients are particularly vulnerable to SARS-CoV-2 infection due to profound immune dysfunction and the prolonged timeline for immune reconstitution, especially in heavily pretreated patients in the early transplantation period (<12 months). Hopefully, encouraging preliminary data suggests that SARS-CoV-2 vaccination reduces the severity of breakthrough COVID-19 infection in this population with mortality <10% [30]. In our cohort, there was no breakthrough infection. Thus, these observations are important when designing preventive measures within the continuing COVID-19 pandemic for vulnerable subgroups of citizens. Especially nowadays, where preventive monoclonal antibodies have become available but are still in limited supply, careful prioritization of people at high risk should be applied. In this context, auto-HSCT per se does not represent a high-risk feature, and these patients should follow the National Vaccination Programs since they elicit adequate AR. On the other hand, hypogammaglobulinemic patients, especially IgA, those on lymphoma or myeloma treatment, especially with monoclonal antibodies, heavily treated patients, and those who are close to HSCT are the ones who are less likely to respond to vaccination and should receive prophylactic monoclonal antibodies against SARS-CoV-2. Another aspect based on these observations is that post-transplant treatment should be given with caution during the pandemic, especially if administered as maintenance. Although the morbidity and mortality of COVID-19 disease have decreased with the newer virus strains, immunocompromised patients may still experience serious complications. Moreover, SARS-CoV-2 infection may lead to delays in treatment with serious consequences in disease outcomes. Even nowadays immunization represents the major intervention against SARS-CoV-2 in patients with hematologic malignancies undergoing auto-HSCT.

## 5. Conclusions

Conclusively, over 80% of patients who have undergone HSCT do elicit antibody responses after vaccination with the BNT162b2 vaccine (Pfizer–BioNTech COVID-19 vaccine, New York, NY, United States-Mainz, Germany) against SARS-CoV-2 with a declining trend over a 3-month period. Treatment given post-transplant and especially anti-CD20, as well as hypogammaglobulinemia, are the major determinants of AR, rather than transplantation itself.

## Figures and Tables

**Table 1:** Patients’ Characteristics.

Characteristic	*N* [%]
Median age in years (range)	56 (19–71)
Median time from HSCT in months (range)	32.9 (6.4–60)
Male	30 [55.6]
Underlying hematologic malignancy	
*Lymphoma*	31 [57.4]
*Hodgkin*	12 [22.2]
*non-Hodgkin*	19 [35.2]
*Multiple myeloma*	19 [35.2]
*Acute leukemia*	4 [7.4]
Type of HSCT	
*Autologous*	50 [92.6]
Treatment at the time of vaccination	24/51 [47.1]
*Anti-CD20 antibody*	5 [10.0]
*Lenalidomide*	11 [22.0]
*Other ^α^*	8 [14.0]
Prior lines of treatment, median (range)	3 (2–8)
Patients with hypogammaglobulinemia	
*IgG levels <700 mg/dL*	11/43 [25.6]
*IgG levels <500 mg/dL*	5/43 [11.6]
*IgA levels <70 mg/dL*	10/40 [25.0]
*IgM levels <40 mg/dL*	21/43 [48.8]
*Any class hypogammaglobulinemia ^β^*	26/43 [60.5]
Measurement of AT post-vaccination	
*Both at 1 month and at 3 months*	47 [87.0]
*At 1 month only*	5 [9.0]
*At 3 months only*	2 [4.0]

*^α^* Other; Brentuximab-Vedotin, Bendamustine, Pomalidomide, Carfilzomib, Daratumomab, Azacitidine. *^β^* IgG levels <500 mg/dL and/or IgA levels <70 mg/dL and/or IgM levels <40 mg/dL. HSCT; hematopoietic stem cell transplantation, AΤ; antibody titers.

**Table 2:** Characteristics of patients with no detectable antibody response.

Pt	Gender/ Age (y)	Disease	Hypo-γ	Time from HSCT to Vaccination (mo)	Tx at Vaccination	No. of Tx Lines Before Vaccination
1	M/68	DLBCL	No	23.90	No	3
2	F/49	HL	No	50.39	No	7
3	M/37	FL	Yes	21.41	Rituximab	4
4	F/62	AITL	Yes	21.87	BV/Benda	3
5 ^α^	F/66	FL	Yes	59.05	Azacitidine	6
6	M/47	MCL	Yes	9.64	Rituximab	3
7	M/67	MCL	Yes	39.15	Rituximab	3
8	F/65	MM	Yes	13.64	Pom/Dex	8
9	F/69	MM	Yes	45.64	Carlf/Dex	8

^α^: Patient auto-transplanted for FL, who developed secondary MDS with monosomy 7 and was receiving azacitidine at the time of vaccination. Pt: patient, y: year-old, Hypo-γ: hypogammaglobulinemia, HSCT: hematopoietic stem-cell transplantation, mo: month, **Tx:** treatment, No: number, M: male, DLBCL: diffuse large B-cell lymphoma, F: female, HL: Hodgkin lymphoma, FL: follicular lymphoma, AITL: angioimmunoblastic lymphoma, BV: brentuximab vedotin, Benda: bendamustine, MCL: mantle-cell lymphoma, MM: multiple myeloma, Pom: pomalidomide, Dex: dexamethasone, Carlf: carfilzomib.

**Table 3:** Distribution of antibody titers at 1- and 3-months post-vaccination.

Antibody Titers Post-Vaccination (U/mL)	1 Month After, %	3 Months After, %
<0.8	19.2	14.3
0.8–19.9	5.8	6.1
20–249.9	13.5	26.5
250–999.9	23.1	22.4
1000–1999.9	13.5	18.4
2000–4999.9	15.4	10.2
≥5000.0	9.6	2.0

**Table 4:** Significant prognostic factors for antibody response.

Factor	N	AR at m_1_		AT at m_1_ (U/mL)		*p*	N	AR at m_3_		AT at m_3_ (U/mL)		*p*
		Pos [%]	*p*	Median	Range			Pos [%]	*p*	Median	Range	
**IgA hypogamma**			0.019			0.02			0.046			0.04
IgA < 70 mg/dL	10	5 [50.0]		5.3	0.4–112		10	6 [60.0]		25.38	0.4–1504	
IgA ≥ 70 mg/dl	29	25 [86.2]		388	0.4–25,000		27	24 [88.8]		269	0.4–7869	
**Any class hypogamma**			0.02			0.018						0.017
Yes	25	16 [64.0]		110	0.4–3419		22	16 [72.7]	0.08	86.7	0.4–2486	
No	17	16 [94.1]		1065	0.4–25,000		17	16 [94.1]		850	0.4–7869	
**Combined IgA hypogamma** **and levels of other Ig classes**			0.04			0.049			0.1			0.06
Group A	15	14 [93.3]		538	0.4–25,000		15	14 [93.3]		703	0.4–7869	
Group B	14	11 [78.5]		277	0.4–3419		12	10 [83.3]		203	0.4–2486	
Group C	10	5 [50.0]		5.3	0.4–1112		10	6 [60.0]		25.3	0.4–1704	
**Time from HSCT to vaccine**			0.6			0.037			1			0.29
<18 months	44	36 [81.8]		64.25	0.4–828		42	36 [85.7]		118	0.4–1704	
≥18 months	8	6 [75.0]		767	0.4–25,000		7	6 [85.7]		339	0.4–7869	
**Treatment**			<0.001			<0.0001			0.006			<0.0001
No treatment	26	24 [92.3]		1488	0.4–25,000		26	24 [92.3]		1193	0.4–7869	
Anti-CD20 antibody	5	1 [20.0]		0.4	0.4–423		5	2 [40.0]		0.4	0.4–97.9	
Other	8	4 [50.0]		9755	0.4–1844		6	4 [66.6]		95.85	0.4–850	
Lenalidomide	10	10 [100.0]		210	10.2–1100		10	6 [60.0]		118.75	4.06–418	
**Previous lines of Tx**			0.137			0.016			0.237			0.026
<3	7	7 [100.0]		2174	282–3591		7	7 [100.0]		1647	118–2486	
≥3	36	27 [75.0]		330	0.4–25,000		35	29 [83.0]		145	0.4–7869	

AR: antibody response, AT: antibody titers, m_1_: one month post-vaccination, m_3_: three months post-vaccination, any class hypogamma: IgG < 500 mg/dL and/or IgA < 70 mg/dL and/or IgM < 40 mg/dL, group A: patients with normal levels of all classes of immunoglobulins; group B: patients with normal IgA levels and lower than normal levels of the other two classes of immunoglobulins (either IgG or IgM or both); group C: IgA hypogammaglobulinemia, irrespectively of the values of the other two immunoglobulin classes, HSCT; hematopoietic stem cell transplantation, Tx: treatment.

**Table 5:** Single-center studies that examined the serologic response of two doses of the BNT162b2 (Pfizer) vaccine against SARS-CoV-2 in hematopoietic stem-cell transplantation recipients.

Study, First Author	Ref.	N	AGE, y (δ), [Range]	HSCT Type	TΙTV, m (δ), [Range]	TMV, d (δ), [Range]	RR [%], (PCO)	AT (δ), [Range]
ATTOLICO	[9]	114	56 [20–71]	BOTH ^α^	NA ^β^	28 [NA] ^γ^	84 (≥50 AU/mL)	4481 [0–104,689]
AUTORE	[10]	58	59 [27–71]	AUTO	8 [0.6–17]	65 [24–214]	67 (>0.8 BAU/mL)	139.533 [0.02–11,097]
CANTI	[11]	37	60 [26–76]	ALLO	31 [5–51]	28 ^δ^	86 (>5 IU/mL)	NA
CHEVALLIER	[12]	112	57 [20–75]	ALLO	22 [3–206]	21.5 [16–35] ^ε^	55	NA
CHIARUCCI	[13]	50	60 [21–72]	BOTH ^στ^	13 [0.2–26]	30	76	282 AU/mL [68–>400]
DHAKAL ^ζ^	[14]	130	65 [25–77]	BOTH ^η^	NA	NA	60	NA
MAMEZ ^θ^	[15]	63	54 [18–78]	ALLO	14 [3–150]	38 [13–98]	76	815 IU/mL [NA]
MATKOWSKA	[16]	65	21 [18–31]	ALLO	126 [36–324]	NA [14–21]	96	NA ^ι^
KIMURA (3-dose vacc)	[17]	122	57 [45–64]	ALLO	d2; 8.3[5.3–22.7] d3; 15.3[9.8–29.4]	NA	89.1 (after 3rd dose)	2nd dose; 125.6 U/mL [2.8–1251] ^κ^ 3rd dose; 10,358 U/mL [673.9–31.753] ^κ^
LE BOURGEOIS	[18]	117	57 [20–75]	ALLO	22 [3–207]	35 [18–77]	83 (>0.8 U/mL)	NA
LECLERC ^λ^	[19]	133 ^μ^	NA	ALLO	NA	NA ^ν^	72 ^ξ^	NA
PINANA ^ο^	[20]	397	59 [18–80]	BOTH	93 [3–763]	21 [15–59]	78	NA
RAM	[21]	66	65 [23–83]	ALLO	32 [3–263]	NA [7–14]	75	178 [0.4–250]
REDJOUL	[22]	88	28 [26–31]	ALLO	23 [3–213]	28 [26–31] ^κ^	78	NA
SALVINI	[23]	64	62 [29–75]	AUTO	25.6 [1.2–58.1]	28 [25–48]	87	747 BAU/mL [101–2018]
SHEM-TOV	[24]	152	58 ^π^ [22–82]^κ^	ALLO	41 [24–77]	28 [8–69] ^κ^	78	NA
TAMARI ^ρ^	[25]	217	66 [25–84]	BOTH ^σ^	36 [17–63] ^κ^	28 and 90	1-m; 61 ^τ^ 3-m; 87	1-m; 479.75 AU/mL [170.4–3658.8] ^κ^ 3-m; 5379 AU/mL [451–15,750] ^ξκ^
WATANABE	[26]	25	55 [23–71]	ALLO	57 [6–147]	NA ^υ^	76	NA
PRESENT STUDY		54	56 [19–71]	BOTH	33 [6–60]	28 and 90	1-m; 80.8 3-m; 85.7	1-m; 480.5 U/mL [0.4–25,000] 3-m; 293 U/mL [0.4–7869]

^α^; autologous; 46%, allogeneic; 54%, ^β^; Patients were stratified in three groups, according to the time elapsed from transplant to vaccination: G1 ≤1 year (19 patients); G2 1–5 years (52 patients); G3 ≥5 years (43 patients), ^γ^; Antibodies were measured 4 weeks after vaccination completion, ^δ^; Antibody levels were quantified at days 21, 28 and 49 after the first dose of vaccination, ^ε^; the interval between the first dose and the serology assay, ^στ^; autologous = 76%, allogeneic = 24%, ^ζ^; Pfizer = 59%, Moderna = 36%, Johnson & Johnson = 5%, ^η^; Autologous = 35%, Allogeneic = 55%, CAR T-cell therapy = 10%, ^θ^; 73% of patients received mRNA-1273 and 27% received BNT162b2, ^ι^; the authors report the geometric mean concentration of antibodies, ^κ^; the range refers to the interquartile range, ^λ^; anti-SARS-CoV-2 antibodies dynamics were quantified at 6 months following vaccination, ^μ^; 47 patients showing titers below 4160 AU/mL 1 month after the second dose, received a third dose 51 ± 21 days after the second dose, ^ν^; mean time: 59 ± 17 (after two vaccine doses), 103 ± 25 (after three vaccine doses) and 184 ± 15 days following the first dose, ^ξ^; had level above 1000 AU/mL (chosen as a threshold being able to neutralize variants of concern), ^ο^; Moderna = 81%, Pfizer = 14%, Astra-Zeneca = 4.3%, Janssen = 0.15%, ^π^; it refers to mean age, ^ρ^; Pfizer = 70%, Moderna = 30%, ^σ^; Autologous = 69%, Allogeneic = 28%, CAR T-cell therapy = 3%, ^τ^; At 1 month after first vaccine dose only 39(18%) patients were tested for response,^υ^; Peripheral blood samples were collected within 7 days prior to the second dose and 14 days (±7 days) after the second dose of BNT162b2. N; number of patients, y; years, δ; mean value, HSCT; hematopoietic stem cell transplantation, TITV; Time interval between transplantation and vaccination, m; month(s), TMV; time point of antibody measurement after second vaccine dose, d; day(s), RR; positivity antibody-response rate, PCO; antibody-response positivity cut-off, AT; antibody titers, BOTH; allogeneic and autologous transplantation, NA; not available, ALLO; allogeneic.

## Data Availability

No new data was created or analyzed in this study. Data is contained within the article.
